# Conserved spike epitopes persist in generalist bat sarbecoviruses

**DOI:** 10.1371/journal.pbio.3004071

**Published:** 2026-10-27

**Authors:** Matthew B. Frieman

**Affiliations:** Center for Pathogen Research, The Department of Microbiology and Immunology, The University of Maryland School of Medicine, Baltimore, Maryland, United States of America

## Abstract

Bat sarbecoviruses differ in the range of mammalian ACE2 receptors they can use, complicating predictions of spillover risk. This Primer comments on two new studies in PLOS Biology that link receptor breadth with antigenic relatedness and reveal conserved spike epitopes targetable by antibodies.

## Teaser

Bat sarbecoviruses differ in the range of mammalian ACE2 receptors they can use, complicating predictions of spillover risk. Two new studies in *PLOS Biology* link receptor breadth with antigenic relatedness and reveal conserved spike epitopes targetable by antibodies.

Coronavirus entry is initiated by the trimeric, membrane-anchored spike protein. Spike binds a cellular receptor, which for many sarbecoviruses is angiotensin-converting-enzyme 2 (ACE2). The amino acid sequence of ACE2 differs across species, and whether spike can bind a cell’s ACE2 can determine host range and spillover potential [1]. Understanding how spike protein evolution shapes animal-to-human spread requires figuring out how the spike–receptor interaction evolves. Evolutionary trade-offs exist between being a spike protein that can bind many different ACE2 proteins and being species-specific. That difference can be exploited to design better therapeutics for future coronavirus outbreaks.

Bats are known to harbor a high diversity of coronaviruses, and betacoronaviruses are found in several bat species, making them important natural reservoirs for these viruses [2]. Within this diversity, sarbecoviruses exist on a spectrum from ‘generalists’, capable of infecting multiple mammalian host species, to ‘specialists’, adapted to narrow host ranges [3]. In this issue of *PLOS Biology*, two complementary immunological and structural studies examine how sarbecovirus receptor usage relates to antigenicity and identify conserved spike epitopes that may be exploited for the development of monoclonal antibodies with broad anti-coronavirus efficacy (Fig 1).

**Fig 1:**
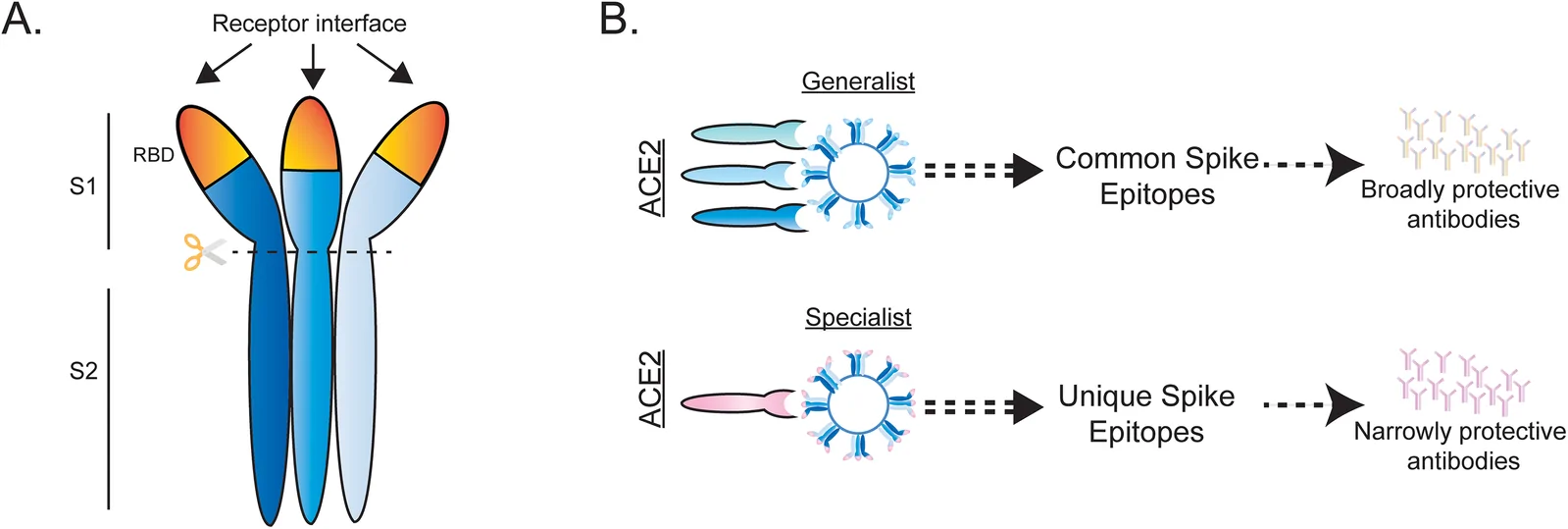
Spike proteins that bind many different ACE2 species reveal common epitopes for therapeutic development. **A.** Diagram of the coronavirus spike protein. The spike protein is divided into S1 and S2 domains, with the S1 domain containing the receptor binding domain (RBD) that interacts with the cognate receptor. The S2 domain is integrated in the virion membrane and allows for extension of the RBD. Cleavage sites between S1 and S2 are critical for spike processing and allow fusion with the plasma membrane of the target cell after receptor binding. **B**. Coronaviruses can be classified as ‘generalists’ or ‘specialists’. Generalists bind many different ACE2 proteins from different mammalian species, while specialists bind species-specific ACE2 proteins. Generalist spike proteins have motifs that allow for binding to various ACE2 alleles, however that reveals antibody epitopes that are bound by more monoclonal antibodies. Specialist spike proteins do not have these common motifs and are bound by fewer monoclonal antibodies in these studies.

First, Thakur and colleagues investigated how different coronavirus spike–receptor interactions can determine the probability of future spillover from bat sarbecoviruses into humans [4]. The authors found that spike proteins from the generalist sarbecoviruses can use many different mammalian ACE2 proteins, with the generalist spike proteins phylogenetically and antigenically related. While the ability to cross species can be a fitness advantage for some viruses and increase potential spread to other mammalian species, their study suggests that this receptor promiscuity can come with an antigenic cost. They showed that sera from convalescent SARS-CoV-2-infected and vaccinated people bind generalist spike proteins better than it does specialist spike proteins because generalist sarbecovirus spikes have more common epitopes among them that human immune systems have seen before. The authors propose that while spillover risk may be increased by *sarbecovirus* lineages that could spread to humans, there is a lower likelihood of this happening because humans may already have antibodies that bind these spike proteins. The common epitopes in the generalist sarbecovirus spike proteins would actually limit the virus’s ability to spread in humans due to pre-existing immunity and reduce their true pandemic potential compared to a totally naïve host.

Upadhyay and colleagues also used the feature of common epitopes to their advantage in identifying monoclonal antibodies that bind these conserved epitopes, potentially allowing for their use as broadly effective therapeutics [5]. The very receptor generalism that could make clade 1 sarbecoviruses a spillover risk because they are able to spread to many mammalian hosts means that the interface of spike to ACE2 has limited structural variability, reducing the sequence variation allowed while still retaining their binding capability to a range of ACE2 proteins. The authors used SARS-CoV-2 convalescent sera to identify antibodies that bind these conserved regions. They found that monoclonal antibodies targeting these epitopes neutralize a range of divergent bat sarbecoviruses. This provides evidence that these epitopes are evolutionarily constrained and, once identified, these epitopes could be used for rational pan-coronavirus vaccine design.

The spike protein is not a rigid structure but, rather, it is dynamic; the RBD can face up or down depending on mutations in the protein sequence and how it interacts with its cognate receptor. Studies have shown that this ability to shift structure translates into how fast the spike protein is cleaved by furin or host proteases and, ultimately, delivers the viral RNA into cells [6,7]. During infection, additional host proteins and localized differences in lipids on the plasma membrane can also interact with the virion to determine the speed of the initial spike–receptor binding [8,9]. The kinetics of a virion binding to its receptor and fusing to the cellular membrane are important because the faster a virion can deposit viral RNA into a cell, the less time antibodies have to bind spike and block virus entry. The immune recognition cost of being a generalist sarbecovirus with the common epitopes, shown by these two manuscripts [4,5], is balanced against the spike–ACE2 binding affinity, suggesting there are many factors that are involved in virus spillover and spread in humans.

Together, these papers identify a potentially important vulnerability in sarbecovirus pandemic potential: the common epitopes found in a subset of sarbecoviruses that allow these viruses to infect a variety of mammalian species can be used as broad targets for therapeutic design. The mechanism of this conservation is an additional intriguing question that can be used to investigate coronavirus evolution. After spike–ACE2 binding and entry, there are other post-entry barriers to viral replication, including host innate immunity, interaction with host proteases and other direct protein–protein interactions that impact virus spillover into mammals. These, combined with the spike conservation found in both of these papers, provide interesting avenues for further research in general coronavirus biology.

Both papers are trying to answer a practical question: which bat coronaviruses could realistically jump into people next, and could we build drugs or vaccines to have at the ready before that happens. Thakur and colleagues find that these viruses fall into two broad camps—generalists that can bind to the ACE2 receptor of many different mammals, and specialists that are picky and mostly stay in their own hosts [4]. The generalists turn out to be the close relatives of SARS-CoV-2. They are the viruses most likely to infect humans or pass through an intermediate animal, but because they look similar to SARS-CoV-2 they are also the ones our existing immunity recognizes best. Antibody-based binding of spike drops off sharply for the more distant branches of the family, so surveillance and countermeasures should not just track the SARS-CoV-2-like viruses. In their paper, Upadhyay and colleagues find a way around this gap [5]. The authors identify a few epitopes on the virus’ spike protein that barely change across the whole family, because the virus cannot alter them without losing viral fitness, and show that antibodies aimed at those spots are able to neutralize both distant bat viruses and the newest SARS-CoV-2 variants [10]. Taken together, the implication is that a pan-sarbecovirus vaccine or stockpiled antibody treatment is a realistic goal if we can aim therapeutics at the conserved, constrained parts of the spike protein rather than the variable spike surface that everyone’s immunity currently relies on.
